# Supplementary material for: Synergistic Effect between the APOE ε4 Allele with Genetic Variants of GSK3B and MAPT: Differential Profile between Refractory Epilepsy and Alzheimer Disease
Source: Int J Mol Sci. 2024 Sep 23;25(18):10228. doi: 10.3390/ijms251810228 (PMC11432663; doi:10.3390/ijms251810228)
Supplement: Supplementary file 1 [file ijms-25-10228-s001.zip › TABLE S1.pdf]

TABLE S1. Study SNPs data

GEN	SNP	CHROMOSOME	CHROMOSOME POSITION	GENE LOCATION	ANCESTRAL ALLELE	POLYMORPHIC ALLELE
**MAPT**	**rs242557**	17	41375548	5’ EXON 1	G	G
**MAPT**	**rs1467967**	17	43986179	5’ EXON 1	A	G
**MAPT**	**rs2471738**	17	44076063	INTRON 9	C	T
**MAPT**	**rs7521**	17	44105395	3’ EXON 14	G	A
**MAPT**	**rs3785883**	17	44054433	INTRON 3	G	G
**HSPA1L**	**rs2227956**	6	31778272	EXON 2	T	C
**HSPA5**	**rs391957**	9	128004024	5’ UTR	C	T
**GSK3**	**rs334558**	3	119813282	5’ UTR(-50)	G	A
**GSK3**	**rs6438552**	3	6438552	INTRON 5	G	A
