# Supplementary material for: Synergistic Effect between the APOE ε4 Allele with Genetic Variants of GSK3B and MAPT: Differential Profile between Refractory Epilepsy and Alzheimer Disease
Source: Int J Mol Sci. 2024 Sep 23;25(18):10228. doi: 10.3390/ijms251810228 (PMC11432663; doi:10.3390/ijms251810228)
Supplement: Supplementary file 1 [file ijms-25-10228-s001.zip › TABLE S2.pdf]

Table S2. Hardy–Weinberg equilibrium in HSP70 and HSP90

Gene	Polymorphism	nHS-TLE n=49	HS-TLE n=79	TA-TLE n=70	Control n=245	AD n=100	AD Control n=106
HSP70	**rs2227965**						
	T	80 (82%)	112 (71%)	130 (93%)	316 (64%)	51 (26%)	40 (19%)
	C	18 (18%)	46 (29%)	10 (7%)	174 (36%)	149 (74%)	172 (81%)
	C/C	5 (10.2%)	14 (17.7%)	1 (1.4%)	71 (29%)	59 (59%)	68 (64.1%)
	T/C	8 (16.3%)	18 (22.8%)	8 (11.4%)	32 (13.1%)	31 (31%)	36 (34%)
	T/T	36 (73.5%)	47 (59.5%)	61 (87.1%)	142 (58%)	10 (10%)	2 (1.9%)
	x^2	7.51	6.57	25.38		6.17	
	p	**0.023**	**0.037**	**<0.00001**		**0.046**	
	EHW p	**0.0045**	**0.00018**	0.29	**<0.0001**	0.068	0.35
	**MODELS**					**[OR(CI), p]**	
	"A/G+A/A" VS G/G					0.245 (0.047 - 1.274), 0.095	1
	"A/G+A/A" & G/G * APOE					---	
	A/A VS "A/G+G/G"					0.978 (0.502 - 1.906), 0949	1
	A/A VS "A/G+G/G" * APOE					0.556 (0.133 - 2.321), 0.42	
	A/G VS "A/A+G/G"					0.755 (0.375 - 1.521), 0.423	1
	A/G VS "A/A+G/G" * APOE					----	
HSP90	**rs391957**						
	C	67 (68%)	86 (54%)	111 (79%)	230 (47%)	21 (0.1%)	5 (2%)

T	31 (32%)	72 (46%)	29 (21%)	260 (53%)	179 (0.9%)	207 (98%)
C/C	30 (61.2%)	35 (44.3%)	50 (71.4%)	88 (36%)	5 (5%)	0 (0%)
C/T	7 (14.3%)	16 (20.3%)	11 (15.7%)	54 (22%)	11 (11%)	5 (4.7%)
T/T	12 (24.5%)	28 (35.4%)	9 (12.9%)	103 (42%)	84 (84%)	101 (95.3%)
x^2	10.91	1.84	29.76		8.6	
p	**0.004**	0.39	**<0.00001**		**0.013**	
EHW p	**<0.0001**	**<0.0001**	**<0.0001**	**<0.0001**	**0.00092**	1
**MODELS**					**[OR(CI), p]**	
"A/G+A/A" VS G/G					1.506 (0.351 - 6.460), 0.581	1
"A/G+A/A" & G/G * APOE					----	
A/A VS "A/G+G/G"					3.104 (0.888 - 10.846), 0.076	1
A/A VS "A/G+G/G" * APOE					1.211 (0.098 - 14.945), 0.882	
A/G VS "A/A+G/G"					1.874 (0.481 - 7.293), 0.365	1
A/G VS "A/A+G/G" * APOE					1.307 (0.096 - 17.756), 0.841	

nHS-TLE; Non hippocampal sclerosis-Temporal lobe epilepsy
HS-TLE; hippocampal sclerosis- Temporal lobe epilepsy
Data are expressed as mean ±SD
