# Supplementary material for: Synergistic Effect between the APOE ε4 Allele with Genetic Variants of GSK3B and MAPT: Differential Profile between Refractory Epilepsy and Alzheimer Disease
Source: Int J Mol Sci. 2024 Sep 23;25(18):10228. doi: 10.3390/ijms251810228 (PMC11432663; doi:10.3390/ijms251810228)
Supplement: Supplementary file 1 [file ijms-25-10228-s001.zip › TABLE S3.pdf]

**Table S3.** Socio-demographic data of patients assessed cognitive

| | nHS-TLE | | HS-TLE | |
|---|---|---|---|---|
| | n=19 | | n=23 | |
| | Males | Females | Males | Females |
| **n** | 8 | 11 | 13 | 10 |
| **Age (years) Mean ±SD** | 43.50±13,17 | 33.45±9.40 | 41.69±10.91 | 37±8.34 |
| **Education ±SD** | 11±3.16 | 9.18±3.06 | 11.23±2.68 | 13.80±3.80 |

nHS-TLE; Non hippocampal sclerosis-Temporal lobe epilepsy
HS-TLE; hippocampal sclerosis- Temporal lobe epilepsy
Data are expressed as mean ±SD
