# Supplementary material for: Synergistic Effect between the APOE ε4 Allele with Genetic Variants of GSK3B and MAPT: Differential Profile between Refractory Epilepsy and Alzheimer Disease
Source: Int J Mol Sci. 2024 Sep 23;25(18):10228. doi: 10.3390/ijms251810228 (PMC11432663; doi:10.3390/ijms251810228)
Supplement: Supplementary file 1 [file ijms-25-10228-s001.zip › TABLE S4.pdf]

TABLE S4. Neuropsychological evaluation protocol

Domain	Test/Subtest
**Orientation**	-Orientation (MoCA)<br>-Orientation (Barcelona Test)
**Atention**	-Digits in progression and regression (Wechsler Memory Scale)<br>-Keys or digits and symbol of WAIS IV<br>-Trail Making Test (TMT)
**Expressive language**	-Repetition<br>-Denomination<br>-Verbal fluency<br>-Subtests of Vocabulary, Comprehension, Information of WAIS-IV
**Impressive language**	-Comprehension of orders (Barcelona Test)
**Written language**	-Barcelona Test
**Gnosias**	-Barcelona test<br>-Incomplete Figures WAIS -IV
**Praxias**	-King's Complex Figure (NAM-3)<br>-Wechsler Intelligence Scale (WAIS-IV)
**Memory**	-Wechsler Memory Scale (WMS)<br>-Neuropsi Attention and Memory (NAM-3)
**Executive Functions**	-INECO, Frontal screening<br>-Torre de Londres<br>- Test de Clasificación de Tarjetas de Wisconsin<br>-Test de Stroop
