# Supplementary material for: Synergistic Effect between the APOE ε4 Allele with Genetic Variants of GSK3B and MAPT: Differential Profile between Refractory Epilepsy and Alzheimer Disease
Source: Int J Mol Sci. 2024 Sep 23;25(18):10228. doi: 10.3390/ijms251810228 (PMC11432663; doi:10.3390/ijms251810228)
Supplement: Supplementary file 1 [file ijms-25-10228-s001.zip › TABLE S5.pdf]

Table S5. Non Hippocampal Sclerosis-Temporal Lobe Epilepsy Follow up

	No alterations		Alterations		*p*
	***ε4 carriers**	***ε4 non-carriers**	***ε4 carriers**	***ε4 non-carriers**	
**Orientation**	4 (21.1 %)	15 (78.9 %)	-	-	-
**Atention**	4 (25.0 %)	12 (75 %)	0 (0 %)	2 (100 %)	0.999
**Expressive language**	1 (20.0 %)	4 (80 %)	3 (21.4 %)	11 (78.6 %)	0.637
**Impressive language**	3 (17.6 %)	14 (82.4 %)	1 (50 %)	1 (50 %)	0.999
**Written language**	3 (18.8 %)	13 (81.3 %)	-	-	-
**Gnosias**	3 (18.8 %)	13 (81.3 %)	-	-	-
**Praxias**	2 (15.4%)	11 (84.6 %)	1 (33.3%)	2 (66.7 %)	-
**Memory**	0 (0%)	3 (100 %)	4 (25.0%	12 (75 %)	1
**Executive Functions**	2 (15.4%)	11 (84.6 %)	2 (33.3%)	4 (66.7 %)	0.222

non-Hipocampal TLE n=19 Average follow-up 52.37 months (17-112)
