# Supplementary material for: Synergistic Effect between the APOE ε4 Allele with Genetic Variants of GSK3B and MAPT: Differential Profile between Refractory Epilepsy and Alzheimer Disease
Source: Int J Mol Sci. 2024 Sep 23;25(18):10228. doi: 10.3390/ijms251810228 (PMC11432663; doi:10.3390/ijms251810228)
Supplement: Supplementary file 1 [file ijms-25-10228-s001.zip › TABLE S6.pdf]

Table S6. Hippocampal sclerosis-Temporal Lobe Epilepsy Follow-up

	No alterations		Alterations		*p*
	*ε4 carriers	*ε4 non-carriers	*ε4 carriers	*ε4 non-carriers	
**Orientation**	3 (15.0 %)	17 (85 %)	0 (0 %)	3 (100 %)	1
**Atention**	2 (10.5 %)	17 (89.5 %)	1 (25.0%)	3 (75 %)	0.574
**Expressive language**	2 (22.2 %)	7 (77.8 %)	1 (7.1 %)	13 (92.9 %)	0.509
**Impressive language**	2 (10.5 %)	17 (89.5 %)	1 (15.0 %)	3 (75 %)	0.99
**Written language**	2 (9.5 %)	19 (90.5 %)	0 (0 %)	1 (100 %)	1
**Gnosias**	2 (10.0 %)	18 (90 %)	0 (0 %)	1 (100 %)	1
**Praxias**	2 (10.0 %)	18 (90 %)	1 (100 %)	0 (0 %)	0.999
**Memory**	0 (0 %)	1 (100 %)	3 (13.6 %)	19 (86.4 %)	0.999
**Executive Functions**	1 (7.1 %)	13 (92.9 %)	2 (22.0 %)	7 (77.8 %)	0.998

HS-TLE *n*=23 Mean follow-up 34.36 months (13-97)
