# Supplementary figures and images for: Synergistic Effect between the APOE ε4 Allele with Genetic Variants of GSK3B and MAPT: Differential Profile between Refractory Epilepsy and Alzheimer Disease
Source: Int J Mol Sci. 2024 Sep 23;25(18):10228. doi: 10.3390/ijms251810228 (PMC11432663; doi:10.3390/ijms251810228)

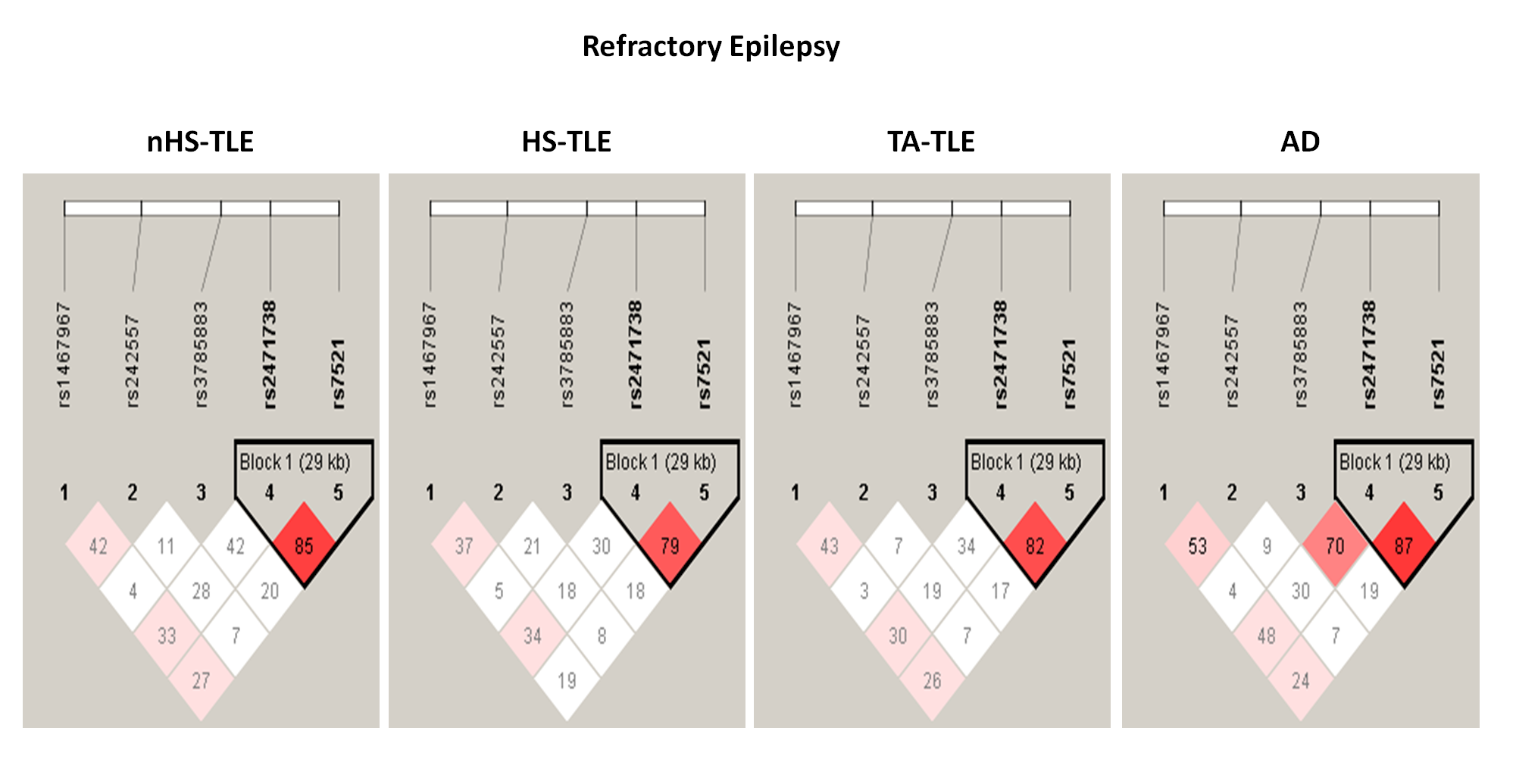

Supplement: Supplementary file 1 [file ijms-25-10228-s001.zip › FIGURE 1S.tif]
